# Repurposing Analysis of Nitroxoline (8-Hydroxy-5-nitroquinoline) as an Antichagasic Compound

**DOI:** 10.3390/ph18081106

**Published:** 2025-07-25

**Authors:** Carlos J. Bethencourt-Estrella, Atteneri López-Arencibia, Isabel M. Calero-Docina, Frieder Fuchs, Patrick Scheid, Jacob Lorenzo-Morales, José E. Piñero

**Affiliations:** 1 Instituto Universitario de Enfermedades Tropicales y Salud Pública de Canarias, Universidad de La Laguna, Avda. Astrofísico Fco. Sánchez, S/N, 38203 La Laguna, Spain; alu0101218717@ull.edu.es (I.M.C.-D.); jmlorenz@ull.edu.es (J.L.-M.); jpinero@ull.edu.es (J.E.P.); 2 Departamento de Obstetricia y Ginecología, Pediatría, Medicina Preventiva y Salud Pública, Toxicología, Medicina Legal y Forense y Parasitología, Universidad de La Laguna, 38203 La Laguna, Spain; 3 CIBER de Enfermedades Infecciosas (CIBERINFEC), Instituto de Salud Carlos III, 28029 Madrid, Spain; 4 Institute for Medical Microbiology, Immunology and Hygiene, University Hospital of Cologne, Medical Faculty, University of Cologne, 50933 Cologne, Germany; frieder.fuchs@uk-koeln.de; 5 Department of Microbiology and Hospital Hygiene, Bundeswehr Central Hospital Koblenz, 56072 Koblenz, Germany; pscheidmedbw@aol.com; 6 Landesuntersuchungsamt Rheinland-Pfalz, 56068 Koblenz, Germany; 7 Department of Biology, Working Group Parasitology and Infection Biology, University of Koblenz, 56070 Koblenz, Germany

**Keywords:** Chagas disease, *Trypanosoma cruzi*, chemotherapy, programmed cell death (PCD), nitroxoline, quinoline

## Abstract

**Background/Objectives:** Chagas disease, caused by the protozoan parasite *Trypanosoma cruzi*, remains a major neglected tropical disease, with over six million cases concentrated, primarily in Latin America. Despite decades of research, treatment continues to rely on two outdated drugs—benznidazole and nifurtimox—both of which exhibit limited efficacy and are associated with severe side effects. In this context, drug repurposing presents a promising strategy to accelerate the development of safer and more effective therapies. Nitroxoline, a hydroxyquinoline compound widely used in Europe to treat bacterial urinary tract infections, has recently garnered attention for its broad-spectrum antimicrobial and anticancer activities. This study evaluated the antitrypanosomal potential of nitroxoline against both epimastigote and intracellular amastigote forms of *T. cruzi*, demonstrating significantly greater efficacy than benznidazole. **Methods:** In addition to its antiparasitic activity, we investigated the mechanism of parasite death and found that nitroxoline induces hallmarks of programmed cell death, including chromatin condensation, mitochondrial membrane depolarization, ATP depletion, reactive oxygen species accumulation, and increased membrane permeability. These cellular events are critical for minimizing host tissue inflammation and suggest a safer therapeutic profile. **Results:** The nitroxoline was shown to induce greater activity than the reference treatment, benznidazole, in addition to triggering events related to apoptotic or silent cell death. **Conclusions:** Given its established clinical use and favorable safety data, nitroxoline emerges as a strong candidate for further investigation as a repurposed treatment for Chagas disease. Future work should focus on in vivo efficacy, pharmacokinetics, and drug delivery strategies to enhance systemic bioavailability.

## 1. Introduction

Chagas disease is caused by the protozoan parasite *Trypanosoma cruzi*. The most common route of transmission is through the bite of infected triatomine bugs, also known as kissing bugs [1]. Triatomine bugs feed on the blood of animals and humans. During feeding, they defecate near the bite site, allowing *T. cruzi* parasites present in the faeces to enter the body through open wounds or mucous membranes. Oral transmission due to contaminated food and drinks or congenital transmission are also possible but less common [1]. Chagas disease is endemic to Latin America, where an estimated 6–8 million cases are concentrated, particularly in Brazil, Colombia, Bolivia, Mexico, and Argentina [2]. A wide variety of mammals, including domestic and wild species such as dogs, armadillos, opossums and rodents, serve as reservoirs for *T. cruzi* [3,4,5].

This parasite has three life stages that must be considered when searching for new treatments. The epimastigote form, which is found and replicates in the insect vector, transforms in the terminal portion of the intestine into the trypomastigote form, which is responsible for initiating infection in the mammalian host. Finally, the amastigote form infects cells within the mammal [2]. These forms of the parasite are adapted to the specific functional requirements of each stage of their life cycle. They exhibit distinctive morphological features, such as the presence of a single mitochondria, which plays a key role in cell survival. Beyond its bioenergetic function, the mitochondria is actively involved in regulating the parasite’s cell cycle [6]. The levels of ATP and reactive oxygen species (ROS) generated by this organelle not only reflect the parasite’s metabolic state, but also act as intracellular signals that modulate essential processes such as differentiation, proliferation, and the stress response [7,8].

There are two main clinical stages of Chagas disease. The acute phase is often asymptomatic but may present mild symptoms, such as the Romaña’s sign. The chronic phase, which can manifest decades later, is characterized by severe complications, including digestive disorders or the characteristic chagasic cardiomyopathy [9,10,11]. In addition to vector-borne transmission, recent years have seen an increase in cases due to non-vector transmission, mainly congenital, from mother to child, as well as through blood transfusions or organ transplants. This type of transmission, along with globalization and migratory movements, has led to a significant increase in cases in non-endemic areas [12,13,14].

Benznidazole and nifurtimox, the only two treatments for Chagas disease, have some limitations, such as incomplete efficacy against the chronic phase of the disease, as well as significant side effects affecting the gastrointestinal and central nervous systems of patients. Despite ongoing efforts to develop new drugs, these treatments, developed more than 50 years ago, remain the first-line options for treating the disease [15]. Therefore, research into potential new drugs that are effective against the parasite and safe for patients’ health is crucial for global health.

**Figure 1:**
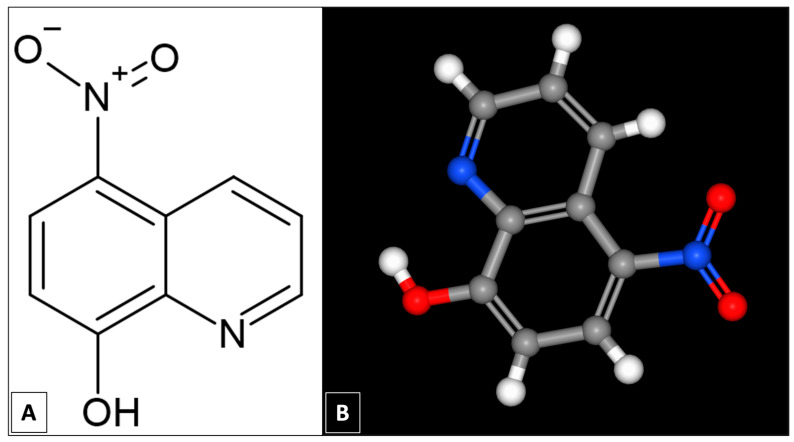
Molecular structure of nitroxoline in 2D (**A**) and 3D (**B**), C_9_H_6_N_2_O_3_, (8-Hydroxy-5-nitroquinoline, MW: 190.156 g/mol). Figure created using ChemSketch 2021.2.1 [26].

Nitroxoline, 8-hydroxy-5-nitroquinoline (Figure 1), is approved in several European countries, including Germany, Croatia, and Poland, for the treatment of bacterial urinary tract infections [16,17,18]. Numerous subsequent studies have also demonstrated other activities related to this hydroxyquinoline. Nitroxoline has demonstrated anticancer activity against prostate cancer [19,20], bladder cancer [21], and glioma [22]. On the other hand, its activity against Mpox [23], SARS-CoV-2 [24], and Epstein-Barr virus [25] has also been demonstrated.

In addition to its activity against viruses, bacteria, and fungi [27,28,29], it has also been shown to be effective against various protozoan parasites. One such parasite is *Acanthamoeba* spp., with IC_50_ values of 0.69 ± 0.01 µM against trophozoites of *A. culbertsoni* [30] and 16.08 ± 0.93 µM against *A. castellanii* [31]. Another protozoan found to be sensitive to this treatment is *Balamuthia mandrillaris*, with an IC_50_ of 5.1 μM [32,33]. Furthermore, activity against another free-living amoeba, *Naegleria fowleri*, has been reported, with IC_50_ values ranging from 1.17 to 1.63 µM [34]. Even the activity against *Trypanosoma brucei* was previously demonstrated (IC_50_ of 0.8 µM) [35].

This oral antibiotic, despite its moderate bioavailability (30–55%), reaches very high urinary concentrations (>100 µg/mL) [36,37], which is key to its clinical efficacy in urinary tract infections. It is primarily eliminated via the kidneys in unchanged form, with a half-life of approximately 2 h [38,39]. Pharmacodynamically, it acts by chelating essential metal ions, thereby interfering with the function of various microbial enzymes, and exhibits activity against gram-positive and gram-negative bacteria, as well as certain fungi and protozoa [16].

It has been previously reported that some pathogens—such as viruses, bacteria, and parasites—share key biological characteristics, including the use of metal-dependent enzymes and susceptibility to oxidative stress. Therefore, compounds like nitroxoline, which chelate essential metal ions and disrupt redox balance, can act against multiple types of pathogens by targeting these common processes [40,41].

On the other hand, previous studies have shown that structurally similar compounds derived from 8-hydroxyquinoline exhibit activity against *T. cruzi*, with potency ranging from 2.17 to 9.42 µM [42].

Given the broad-spectrum antimicrobial properties of nitroxoline, the well-known sensitivity of trypanosomatids to chelating compounds, and its well-established safety profile [43,44], we proposed investigating its activity against the protozoan *T. cruzi*. Additionally, the nature of the cell death induced by nitroxoline in the parasite was examined by studying various cellular and physiological events to determine whether these events triggered programmed cell death, as this silent death process would avoid the inflammatory and immune responses associated with necrotic cell death.

## 2. Results

### 2.1. Biological Activity Results

The results of activity against both replicative intracellular (amastigote) and extracellular (epimastigote) forms of *T. cruzi* (Y strain) are included in Table 1.

Nitroxoline exhibited strong in vitro antitrypanosomal activity, significantly outperforming benznidazole. Against the epimastigote form, nitroxoline had an IC_50_ of 3.00 ± 0.44 µM, compared to 6.92 ± 0.77 µM for benznidazole. For the amastigote form, the difference was comparable (1.24 ± 0.23 µM vs. 2.67 ± 0.39 µM), indicating that nitroxoline is more than twice as potent against both life stages of the parasite.

### 2.2. Mechanisms of Cell Death

In order to understand the type of cell death induced by this compound in parasites, several studies were conducted to investigate the presence of events associated with programmed cell death. These events include increased plasma membrane permeability, ATP depletion, and the accumulation of reactive oxygen species (ROS) [45,46,47,48].

#### 2.2.1. Chromatin Condensation Analysis

**Figure 2:**
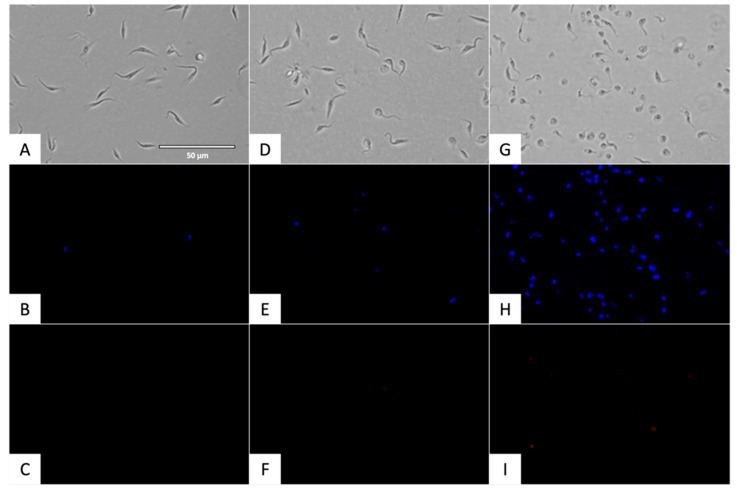
Chromatin condensation detection using Vybrant™ Apoptosis Assay Kit. Results after 24 h of incubation against epimastigote stage of *T. cruzi*. Images were taken using EVOS M5000 Cell Imaging System 1.5.1500.493 (40×). (**A**) Parasites without treatment in visible channel; (**B**) Parasites without treatment in DAPI channel; (**C**) Parasites without treatment in RFP channel; (**D**) Parasites treated with IC_90_ benznidazole in visible channel; (**E**) Parasites treated with IC_90_ benznidazole in DAPI channel; (**F**) Parasites treated with IC_90_ benznidazole in RFP channel; (**G**) Parasites treated with IC_90_ nitroxoline in visible channel; (**H**) Parasites treated with IC_90_ nitroxoline in DAPI channel; (**I**) Parasites treated with IC_90_ nitroxoline in RFP channel. Scale-bar: 50 μm.

**Figure 3:**
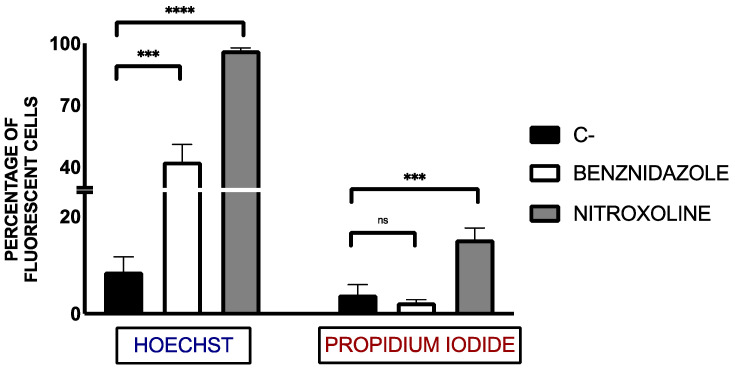
Totals of the percentage of fluorescent cells in the DAPI channel (Hoechst reagent) and the RFP channel (PI reagent). The parasites were counted manually using the EVOS FL Cell Imaging System software. Benznidazole was used as reference treatment. The assays were performed in duplicate on three different days using a Tukey’s test in GraphPad PRISM^®^ 10.1.1 software to assess statistical differences between means relative to the negative control (C−) (ns: no significant; ***, *p* < 0.001; ****, *p* < 0.0001).

Chromatin condensation, a hallmark of programmed cell death, was assessed using the Vybrant™ Apoptosis Assay Kit and can be seen in Figure 2 and Figure 3 Hoechst staining (blue) was used to visualize chromatin condensation in the DAPI channel, while dead cells were labeled with propidium iodide (PI) (red) in the RFP channel.

Nitroxoline treatment led to a significant increase in chromatin condensation, a hallmark of programmed cell death. This effect was comparable to or exceeded that induced by benznidazole, indicating activation of apoptotic-like pathways.

Quantification of fluorescent cells was performed using the EVOS FL Cell Imaging System software. Cells were manually counted across the three fluorescence channels to determine the percentage of apoptotic cells relative to the total population. Statistical analysis was conducted using a Tukey test to compare treated groups with the negative control (untreated).

#### 2.2.2. Mitochondrial Membrane Potential Analysis

**Figure 4:**
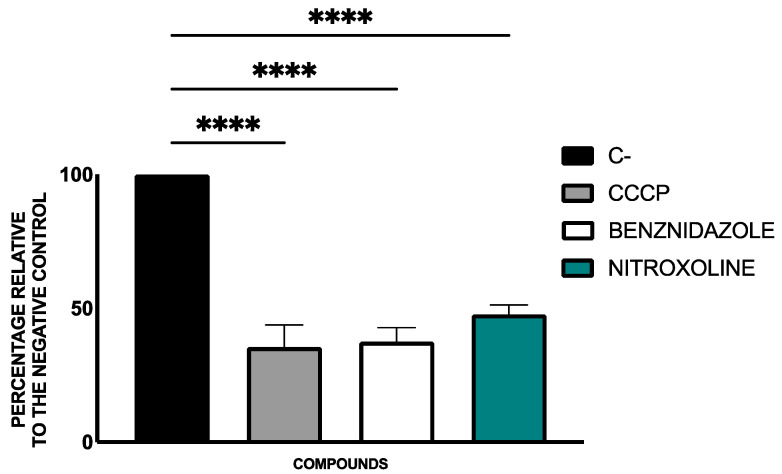
Mitochondrial membrane potential alterations presented as percentage relative to negative control (C−). Benznidazole was used as the reference treatment and Carbonyl cyanide m-chlorophenyl hydrazone (CCCP) as positive control. The assays were performed in duplicate on three different days using a Tukey’s test in GraphPad PRISM^®^ 10.1.1 software to assess statistical differences between means relative to the negative control (****, *p* < 0.0001).

The alterations in the mitochondrial membrane potential were evaluated with the JC-1 Mitochondrial Membrane Potential Assay Kit^®^. The ratios of J-monomers (green fluorescence) to J-aggregates (red fluorescence) were determined and calculated using the EnSpire Multimode Plate Reader^®^ and included in Figure 4.

Nitroxoline caused substantial disruption of the mitochondrial membrane potential, as measured by the JC-1 assay. This depolarization was statistically significant and in line with programmed cell death mechanisms, suggesting mitochondrial involvement in the drug’s mode of action.

#### 2.2.3. ATP Levels Analysis

**Figure 5:**
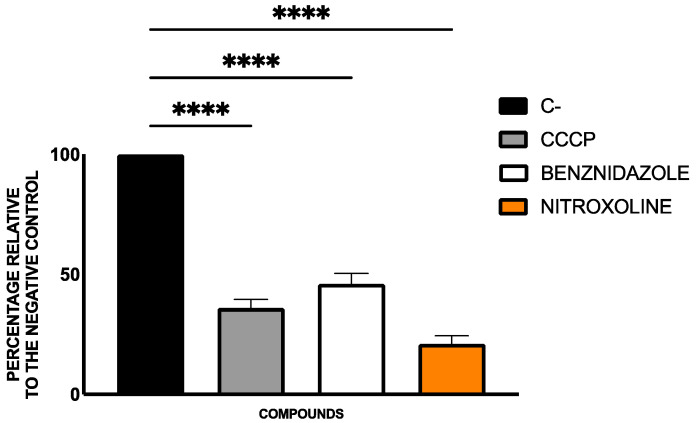
ATP levels represented as percentage relative to negative control (C−). Benznidazole was included as reference treatment, and sodium azide as positive control. The assays were performed in duplicate on three different days using a Tukey’s test in GraphPad PRISM^®^ 10.1.1 software to assess statistical differences between means relative to the negative control (****, *p* < 0.0001).

Cellular ATP levels were quantified using the CellTiter-Glo^®^ Luminescent Cell Viability Assay. The results, expressed as a percentage of ATP relative to the untreated control, were presented in Figure 5.

Treatment with nitroxoline led to a significant decrease in ATP levels in *T. cruzi*, indicating mitochondrial dysfunction and energy depletion. This effect was consistent with the results observed on the mitochondrial membrane potential depletion and matched the results seen with known inhibitors like CCCP.

#### 2.2.4. Plasma Membrane Permeability Analysis

Another characteristic feature of programmed cell death is the alteration of the plasma membrane while maintaining parasite integrity, without complete membrane disruption. In this study, we aimed to detect fluorescence indicative of plasma membrane alteration, as well as to assess the preservation of cell structure at the morphological level [45].

**Figure 6:**
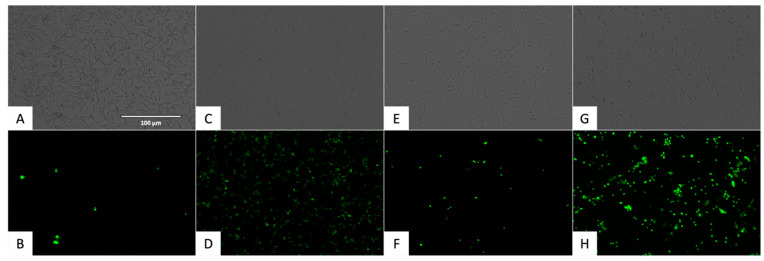
Plasmatic membrane permeability analysis. Images were captured using an EVOS FL Cell Imaging System (40×). Scale-bar: 50 µm. (**A**) Untreated parasites in visible channel; (**B**) Untreated parasites in GFP channel; (**C**) Parasites treated with triton 0.1% in visible channel; (**D**) Parasites treated with triton 0.1% in GFP channel; (**E**) Parasites treated with benznidazole in visible channel; (**F**) Parasites treated with benznidazole, reference treatment; (**G**) Parasites treated with nitroxoline in visible channel. (**H**) Parasites treated with nitroxoline in GFP channel.

Plasma membrane permeability was assessed using SYTOX^®^ Green nucleic acid stain, which selectively enters cells with compromised membranes. Fluorescence images in Figure 6 illustrate membrane permeability alterations.

Nitroxoline increased membrane permeability in *T. cruzi*, suggesting a loss of membrane integrity typical of late-stage programmed cell death. The response was similar to that seen with benznidazole and the positive control (Triton X-100).

**Figure 7:**
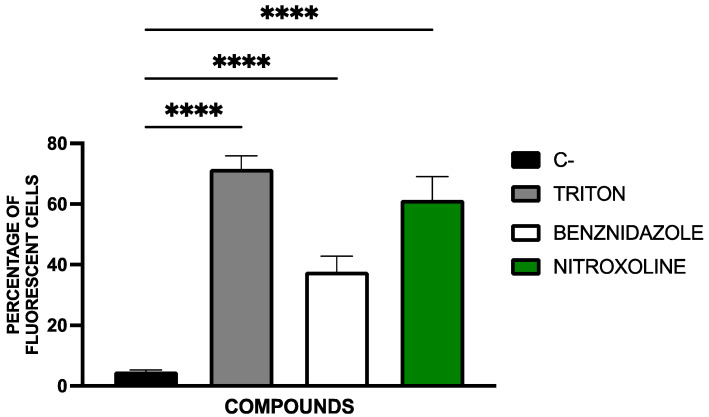
Results of the cell count as a percentage of fluorescent cells. The parasites were counted using the EVOS FL Cell Imaging System software. Benznidazole was used as reference treatment and triton 0.1% as positive control. The assays were performed in duplicate on three different days using a Tukey’s test in GraphPad PRISM^®^ 10.1.1 software to assess statistical differences between means relative to the negative control (****, *p* < 0.0001).

Fluorescent cell quantification was performed using the EVOS FL Cell Imaging System software. The percentage of fluorescent cells relative to the total cell population is presented in Figure 7. Statistical comparisons were conducted using a Tukey test against the untreated control.

#### 2.2.5. Accumulation of Reactive Oxygen Species Analysis

**Figure 8:**
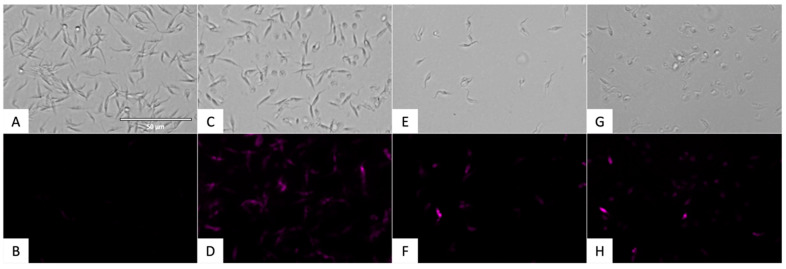
Accumulation of reactive oxygen species. Images were captured using an EVOS FL Cell Imaging System (40×). Scale-bar: 50 µm. (**A**) Untreated parasites in visible channel; (**B**) Untreated parasites in Cy5 channel; (**C**) Parasites treated with H_2_O_2_ in visible channel; (**D**) Parasites treated with H_2_O_2_ in Cy5 channel; (**E**) Parasites treated with benznidazole in visible channel; (**F**) Parasites treated with benznidazole in Cy5 channel; (**G**) Parasites treated with nitroxoline in visible channel. (**H**) Parasites treated with nitroxoline in Cy5 channel.

**Figure 9:**
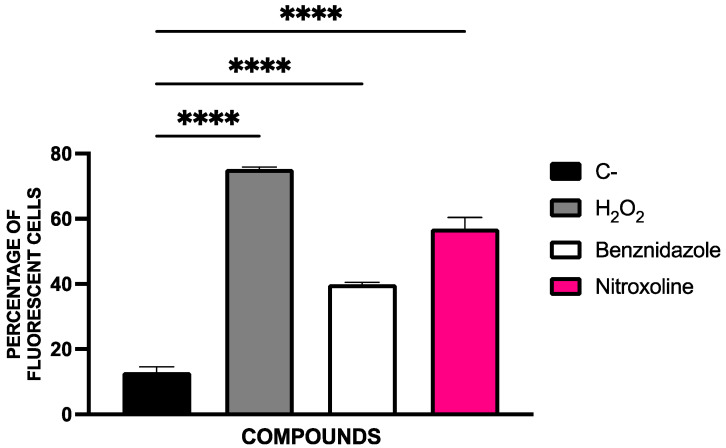
Percentage of fluorescent cells counted. The parasites were counted using the EVOS FL Cell Imaging System software. Benznidazole was used as reference treatment and H_2_O_2_ as positive control. The assays were performed in duplicate on three different days using a Tukey’s test in GraphPad PRISM^®^ 10.1.1 software to assess statistical differences between means relative to the negative control (****, *p* < 0.0001).

Reactive oxygen species (ROS) accumulation in *T. cruzi* was detected using CellROX^®^ Deep Red Reagent and is depicted in Figure 8 and Figure 9. Fluorescent ROS accumulation appears as pink fluorescence in Figure 8.

Nitroxoline significantly increased ROS accumulation in the parasite cytoplasm, as shown by the red fluorescence. This oxidative stress likely contributes to mitochondrial damage and cell death, reinforcing the conclusion that nitroxoline triggers a programmed cell death response.

Quantification of ROS-positive cells was conducted using the EVOS FL Cell Imaging System software. The proportion of fluorescent cells relative to the total population is shown in Figure 9. Statistical comparisons were performed using a Tukey test against the untreated control group.

## 3. Discussion

Previous reports on nitroxoline’s activity against various pathogens suggested its potential effectiveness against *T. cruzi*. Additionally, its documented activity against other protozoa, such as *Acanthamoeba* spp. [30,31], *Balamuthia mandrillaris* [32,33], and *Naegleria fowleri* [34], further supports this hypothesis.

Our results indicate that nitroxoline exhibits strong activity against *T. cruzi*, with potency exceeding that of benznidazole, the current standard treatment. Specifically, nitroxoline demonstrated over twice the efficacy of benznidazole against both the epimastigote form (IC_50_ = 3.00 ± 0.44 vs. 6.92 ± 0.77 μM for benznidazole) and the amastigote form (IC_50_ = 1.24 ± 0.23 vs. 2.67 ± 0.39 μM for benznidazole).

This activity confirms that nitroxoline also exhibits potential activity against *T. cruzi* (1.24–3 µM). These results are comparable to those obtained in previous studies against other protozoa, such as *Naegleria fowleri* (1.17–1.63 µM) [34] and *T. brucei* (0.8 µM) [35]. Moreover, the results from this study represent an improvement over other compounds with a similar structure to 8-hydroxyquinoline (2.17–9.42 µM) [42].

Since nitroxoline acts effectively on the *T. cruzi* protozoan, it was essential to determine what type of death it induced in the parasite, whether it was necrotic cell death, which could trigger severe inflammatory responses in patients, or programmed cell death, which would lead to silent death of the parasite. To this end, we performed multiple analyses of the changes induced in different parasite organelles, such as the plasma membrane, nucleus, or mitochondria, in order to confirm that nitroxoline promotes programmed cell death in parasites [45,46]. Previous studies indicate that trypanosomatids exhibit hallmark features of apoptotic cell death, including chromatin condensation, a defining characteristic of programmed cell death. Additionally, disruption of mitochondrial membrane potential is particularly critical in kinetoplastids, which rely on a single mitochondrion for survival. Other key indicators of programmed cell death include altered plasma membrane permeability, ATP depletion, and the accumulation of reactive oxygen species [45,46,47,48,49]. Our findings confirm that nitroxoline treatment leads to chromatin condensation, mitochondrial membrane potential disruption, reactive oxygen species accumulation, plasma membrane permeability changes, and ATP level fluctuations in *T. cruzi*. These cellular events strongly indicate that nitroxoline induces programmed cell death, thereby minimizing the risk of inflammatory responses and associated side effects in treated patients. In addition to its ability to induce programmed cell death, multiple studies suggest that nitroxoline is safe for therapeutic use [43,44]. A key advantage of drug repurposing is the pre-established safety profile of approved drugs, streamlining their potential clinical application [50,51].

However, it should also be mentioned that this study has some limitations, which are discussed below. Current knowledge of nitroxoline’s pharmacokinetics is primarily based on older studies, as recently reviewed [36]. The unconjugated form of nitroxoline, tested here, is rapidly excreted via the kidneys, resulting in high urinary concentrations but low plasma levels due to its rapid clearance [52]. This should be taken into account when discussing repurposed use of nitroxoline to treat systemic infections such as Chagas disease. On the other hand, there are reports of in vivo therapeutic success outside the urinary tract from animal models and human case studies [22,33], indicating the need for further research on nitroxoline distribution. Treatment perspectives may also be based on targeted drug delivery of nitroxoline, which has been investigated with promising results in cancer research [53,54] and proposed by other groups to overcome treatment limitations, such as with drug-resistant tuberculosis [55]. With respect to the limited number of parasites in our study, the antitrypanosomal activity demonstrated here should also be confirmed with a larger set of *Trypanosoma* spp. to confirm our findings and gain further knowledge about potential treatment opportunities for other diseases caused by these parasites. Overall, the results obtained in this study are consistent with previous studies demonstrating the antitrypanosomatid activity of various quinoline-derived compounds [56,57,58], further confirming that nitroxoline is also among the quinoline derivatives active against *T. cruzi*.

## 4. Materials and Methods

### 4.1. Compounds

The nitroxoline powder (8-Hydroxy-5-nitroquinoline, MW: 190.156 g/mol) was obtained from the manufacturer (Rosen Pharma GmbH, St. Ingbert, Germany). The compound was dissolved at 40 mg/mL in dimethyl sulfoxide (DMSO) and stored at −20 °C.

The benznidazole powder (*N*-Benzyl-2-nitro-1*H*-imidazole-1-acetamide, MW: 260.25 g/mol) was obtained from the manufacturer (Merck KGaA, Darmstadt, Germany). The compound was also dissolved at 40 mg/mL in dimethyl sulfoxide (DMSO) and stored at −20 °C.

### 4.2. Cultures

To perform the extracellular activity assays, epimastigote stage of *T. cruzi* (Y strain) was used. The parasites were cultured in Liver Infusion Tryptose (LIT) with 10% of Foetal Bovine Serum (FBS) at 26 °C. For the intracellular assays, in addition to the parasites, murine macrophages (J774A.1) in Dulbecco’s Modified Eagle Medium (DMEM) supplemented with a 10% of FBS were used, cultured at 37 °C with a 5% CO_2_ atmosphere.

### 4.3. Activity Against Epimastigote Stage

The activity against the epimastigote form of the parasites was expressed as inhibitory concentration 50 (IC_50_). To obtain the IC_50_ against epimastigote stage of the parasites, a colorimetric assay based on the alamarBlue Cell Viability Reagent^®^ (ThermoFisher Scientific, Waltham, MA, USA) reagent was used. In 96-well plates, serial dilutions of nitroxoline (100, 50, 25, 12.5, 6.25, 3.125, 1.56, 0.78, 0.39, 0.19, 0 µg/mL) in LIT were added in a final volume of 100 µL, after which another 100 µL of 10^6^ parasites/mL was added. Finally, 10% of alamarblue reagent was added (20 µL).

After 72 h of incubation at 26 °C, the fluorescence was measured (544 nm excitation, 590 nm emission) using the EnSpire Multimode Plate Reader^®^ (PerkinElmer, Thermo Fischer Scientific, Madrid, Spain). The IC_50_ was calculated with the GraphPad Prism 10.1.1 [59].

### 4.4. Activity Against Amastigote Stage

The IC_50_ against the intracellular form of the parasites, namely the amastigote stage, was determined using the same alamarBlue-based colorimetric method. First, 10^4^ macrophages per well were seeded in 96-well plates. After ensuring complete cell adherence (at least 2 h), 5 × 10^4^ *T. cruzi* parasites were added per well from an aged (6 days) epimastigote culture to ensure a high presence of trypomastigote forms. In order to ensure infection, parasites were added in excess, achieving a macrophage-to-parasite ratio of at least 1:5.

After 24 h, after allowing parasite internalization, the wells were washed to remove uninternalized parasites and then serial dilutions of nitroxoline were added. After a further incubation period of 24 h, the wells were emptied and 30 µL of 0.05% sodium dodecyl sulphate (SDS) was added to lyse the macrophages. This incubation with SDS was only allowed to act for 30 s to damage the macrophage membranes, and then epimastigote culture medium was rapidly added to a final volume of 200 µL, stopping the SDS activity, and promoting the growth of the surviving parasites inside the macrophages. From this point on, the growth conditions of the plate were modified, as the macrophages were being incubated in their culture medium (DMEM), in 37 °C and 5% CO_2_, and they were then transferred to epimastigote culture medium (LIT) and 26 °C. Finally, 10% alamarBlue was added and, after 72 h incubation, fluorescence was measured to determine the IC_50_ [60].

### 4.5. Mechanisms of Cell Death

#### 4.5.1. Chromatin Condensation Analysis

To assess chromatin condensation, the Vybrant^®^ Apoptosis Assay Kit No. 5 (ThermoFisher Scientific, Waltham, MA, USA) was used. Parasites (10^5^ epimastigotes) were incubated with the IC_90_ of nitroxoline and benznidazole for 24 h, followed by centrifugation (3000 rpm, 10 min, 4 °C). The treated cells were then resuspended in 50 µL of buffer. Hoechst (5 µg/mL) and propidium iodide (PI, 1 µg/mL) were added, and after 20 min of incubation at 26 °C, images were captured using the EVOS^®^ FL Cell Imaging System (ThermoFisher Scientific, Waltham, MA, USA). The DAPI light cube was used to visualize Hoechst 33342, which stains condensed chromatin blue (excitation 350 nm/emission 461 nm), while the RFP light cube was used for PI, which stains dead cells red (excitation 535 nm/emission 617 nm) [61].

#### 4.5.2. Mitochondrial Membrane Potential Analysis

To evaluate changes in mitochondrial membrane potential, the JC-1 Mitochondrial Membrane Potential Assay Kit^®^ (Cayman Chemical, Ann Arbor, MI, USA) was utilized. The kit contains JC-1, a cationic molecule that accumulates in the mitochondrial matrix when the mitochondrial membrane potential is normal (high). Due to its high local concentration, JC-1 forms aggregates that emit red fluorescence. In contrast, if the mitochondrial membrane potential is altered or decreased, the attraction to JC-1 is reduced, meaning that it does not accumulate sufficiently to form aggregates and remains in its monomeric form, which emits green fluorescence. After the parasites (10^5^ epimastigotes) were treated with the IC_90_ of nitroxoline and benznidazole for 24 h, they were centrifuged (3000 rpm, 10 min, 4 °C), resuspended in 50 µL of buffer, and transferred to a black 96-well plate. Following the manufacturer’s instructions, 5 µL of the kit solution was added to each well, and after 30 min of incubation, fluorescence was measured using the EnSpire Multimode Plate Reader^®^ (PerkinElmer). Results were expressed as the ratio of J-monomers (green fluorescence, excitation 540 nm/emission 470 nm) to J-aggregates (red fluorescence, excitation 485 nm/emission 535 nm). A reference treatment (benznidazole) and a positive control (carbonyl cyanide m-chlorophenyl hydrazone, CCCP, 100 μM for 3 h) were also included [62].

#### 4.5.3. ATP Levels Analysis

To measure ATP levels in the parasites, the CellTiter-Glo^®^ Luminescent Cell Viability Assay (Promega, Madison, WI, USA) was employed. After treating the epimastigotes (10^5^) for 24 h with the IC_90_ of nitroxoline and benznidazole, they were centrifuged (3000 rpm, 10 min, 4 °C), resuspended in 25 µL of buffer, and transferred to a white 96-well plate. Following the manufacturer’s instructions, 25 µL of the kit solution was thoroughly mixed with the cells (2 min with vigorous shaking). After 10 min of incubation, luminescence was measured using the EnSpire Multimode Plate Reader^®^ (PerkinElmer). Benznidazole served as reference treatment, and sodium azide (NaN_3_, 20 mM for 3 h) was used as a positive control. Results were expressed as a percentage relative to the negative control (non-treated cells) [63].

#### 4.5.4. Plasmatic Membrane Permeability Analysis

To assess the integrity of the plasma membrane and its permeability, the SYTOX^®^ Green nucleic acid stain fluorescent dye (ThermoFisher Scientific, Waltham, MA, USA) was used. This dye can only penetrate cells with compromised membranes, emitting green fluorescence.

For the assay, the epimastigotes (10^5^) treated with the IC_90_ of nitroxoline for 24 h were centrifuged (3000 rpm, 10 min, 4 °C) and resuspended in 50 µL of buffer. The kit was applied at 1 µM, according to the manufacturer’s instructions. After 15 min of incubation, fluorescence images were captured using the EVOS^®^ FL Cell Imaging System (ThermoFisher Scientific, Waltham, MA, USA) with the GFP light cube (excitation 504 nm/emission 523 nm). Benznidazole (IC_90_) was added as a reference treatment and Triton 0.1% as a positive control [64].

#### 4.5.5. Accumulation of Reactive Oxygen Species Analysis

To detect the presence of reactive oxygen species (ROS), the CellROX^®^ Deep Red Reagent (ThermoFisher Scientific, Waltham, MA, USA) was used. This kit contains a reagent that emits fluorescence upon oxidation by ROS.

For the assay, epimastigotes (10^5^) treated with the IC_90_ of the compounds for 24 h were centrifuged (3000 rpm, 10 min, 4 °C) and resuspended in 50 µL of buffer. The reagent was incubated at 5 µM for 30 min, and images were captured using the EVOS^®^ FL Cell Imaging System (ThermoFisher Scientific, Waltham, MA, USA). Benznidazole (IC_90_) was used as a reference treatment, along with a positive control, hydrogen peroxide (H_2_O_2_, 600 mM for 30 min) [65].

### 4.6. Statistical Analysis

The studies of activity were performed in duplicate on three different days using non-linear regression [Inhibitor] vs. response—Variable slope (four parameters) by non-linear regression analysis with 95% confidence, using the GraphPad Prism 10.1.1 statistical software. Furthermore, for the mechanisms of cell death studies, the assays were performed in duplicate on three different days using a Tukey’s test, considering significant values of *p* < 0.05. The ANOVA analysis was also developed in the GraphPad Prism 10.1.1.

## 5. Conclusions

This study demonstrates that nitroxoline, a well-established antimicrobial agent, exhibits potent in vitro activity against *T. cruzi*, the causative agent of Chagas disease. Its efficacy surpassed that of benznidazole, the current first-line treatment, against both extracellular and intracellular forms of the parasite. In addition, nitroxoline triggers multiple programmed cell death processes, such as chromatin condensation, alteration of mitochondrial membrane potential, ATP depletion, accumulation of reactive oxygen species, and alteration of plasma membrane permeability. This mode of action is particularly advantageous as it can reduce host inflammatory responses typically associated with necrotic death of the parasite.

Importantly, nitroxoline’s long-standing clinical use and well-characterized safety profile make it a compelling candidate for drug repurposing in the treatment of Chagas disease. Although pharmacokinetic limitations of the compound, such as its rapid renal excretion, may hinder its systemic application, recent studies and new methods of drug delivery suggest possible avenues to overcome this obstacle.

Overall, the evidence supports the repositioning of nitroxoline as a promising antichagasic agent. However, further investigations are necessary to evaluate its in vivo efficacy, optimize its pharmacological profile, and assess its therapeutic potential in broader *Trypanosoma* species. Expanding this line of research could contribute significantly to the development of safer and more effective treatments for Chagas disease, a condition that continues to impact millions globally.

## Figures and Tables

**Table 1:** Presents the IC_50_ values (mean ± standard deviation, in µM) for nitroxoline against *T. cruzi* intracellular and extracellular forms. Benznidazole was included as a reference treatment. The studies of activity were performed in duplicate in three different days using non-linear regression [Inhibitor] vs. response—Variable slope (four parameters) by non-linear regression analysis with 95% confidence, using the GraphPad Prism 10.1.1 statistical software.

**Activity Against Epimastigote Forms (IC_50_ µM)**			
Nitroxoline	3.00 ± 0.44	Benznidazole	6.92 ± 0.77
**Activity Against Amastigote Forms (IC_50_ µM)**			
Nitroxoline	1.24 ± 0.23	Benznidazole	2.67 ± 0.39

## Data Availability

The data supporting the conclusions of this article is contained within the article.

## Abbreviations

The following abbreviations are used in this manuscript:

DMSO	Dimethyl sulfoxide
LIT	Liver Infusion Tryptose
FBS	Foetal Bovine Serum
DMEM	Dulbecco’s Modified Eagle Medium
IC_50_	Inhibitory concentration 50
SDS	Sodium dodecyl sulfate
PI	Propidium iodide
CCCP	Carbonyl cyanide m-chlorophenyl hydrazone
IC_90_	Inhibitory concentration 90
ROS	Reactive oxygen species
